# Age Inclusive Strategies to Address Mid-Career Faculty Perceptions

**DOI:** 10.1093/geroni/igaf122.1272

**Published:** 2025-12-31

**Authors:** Allyson Graf, Darcey Powell, Deanne Buffalari, Meagan Patterson, John Edlund, Sadie Elder, Crystal Quillen

**Affiliations:** Northern Kentucky University, Highland Heights, Kentucky, United States; Texas A&M University, Corpus Christi, Corpus Christi, Texas, United States; Westminster College, New Wilmington, Pennsylvania, United States; University of Kansas, Lawrence, Kansas, United States; Rochester Institute of Technology, Rochester, New York, United States; Working Wardrobe, High Point, North Carolina, United States; Rutgers Institute for Teaching, Innovation, & Inclusive Pedagogy, New Brunswick, New Jersey, United States

## Abstract

Given a dearth of research and programming targeting mid-career faculty, higher education institutions are largely not responsive to variable career stage needs or age-related changes. The experiences and perceptions of mid-career faculty interpreted through the lens of promoting age-inclusivity can guide institutions in strengthening support at mid-career and beyond. In a sample of self-identified mid-career faculty (N = 94, Mage=45.67, SD = 6.48), 73.9% considered leaving their institution; reasons, which were rated from not at all (1) to to a great extent (3), included reducing stress (M = 2.41, SD = 0.67), institutional concerns (M = 2.37, SD = 0.66), increasing salary (M = 2.24, SD = 0.75), and seeking a supportive work environment (M = 2.04, SD = 0.76). Despite a high percentage of participants considering a move, they also indicated on a 7-point Likert type scale that they were somewhat unlikely to leave in the next three years (M = 2.75, SD = 1.64). We examined psychosocial factors including feeling valued, respected, excluded, isolated, belonging, and defeated within one’s department, institution, and discipline as well as career satisfaction across multiple domains. Isolation, respect, and overall satisfaction with current position were significantly associated with both consideration and likelihood of leaving one’s institution. Isolation, especially within the context of the broader institution, was higher among those who considered leaving (p=.001, g=-0.837) and was also associated with a higher likelihood of leaving within the next three years (r=.31, p=.006). Additional associations will be highlighted for discussion. Strategies for enhancing age-inclusivity among personnel, such as mentorship and recognition, may combat mid-career isolation and bolster feelings of respect. These will be discussed further during the presentation.

